# Supplementary material for: Antibiotic consumption and medication cost in diabetic patients: Insights from Iran health insurance organization (IHIO) claims data
Source: PLoS One. 2026 Feb 27;21(2):e0343090. doi: 10.1371/journal.pone.0343090 (PMC12948126; doi:10.1371/journal.pone.0343090)
Supplement: S1 Table — (DOCX) [file pone.0343090.s001.docx]

**Supporting information**

**S1 Table. The percentage of included participants from different provinces in the study.**

|  | **All** | **No Antibiotic** | **Q1** | **Q2** | **Q3** | **Q4** |
| --- | --- | --- | --- | --- | --- | --- |
| **Antibiotic** | 7.47 (7.46-7.47) | 0.00 (0.00-0.00) | 3.95 (3.94-3.97) | 5.97 (5.96-5.98) | 7.73 (7.72-7.74) | 10.22 (10.21-10.23) |
| **Glucose-lowering drugs** | 7.79 (7.79-7.80) | 14.54 (14.51-14.57) | 11.19 (11.17-11.21) | 9.34 (9.33-9.36) | 7.67 (7.66-7.68) | 5.08 (5.08-5.09) |
| **Total** | 99859824 | 6426526 | 12704037 | 17543904 | 22255883 | 40929474 |
