# Supplementary material for: Antibiotic consumption and medication cost in diabetic patients: Insights from Iran health insurance organization (IHIO) claims data
Source: PLoS One. 2026 Feb 27;21(2):e0343090. doi: 10.1371/journal.pone.0343090 (PMC12948126; doi:10.1371/journal.pone.0343090)
Supplement: S2 Table — A10A: Insulins and Analogues, A10BA: Biguanides, A10BB: Sulfonylureas, A10BD: Combinations of oral blood glucose lowering drugs, A10BF: Alpha glucosidase inhibitors, A10BG: Thiazolidinediones, A10BJ: Glucagon-like peptide-1 (GLP-1) analogues, A10BX: Other blood glucose lowering drugs, excl. insulins. (DOCX) [file pone.0343090.s002.docx]

**Supporting information**

**S2 Table. Percentage of dominant diabetes treatment regimens for antibiotic groups.**

|  | **No antibiotic** | | **Q1** | | **Q2** | | **Q3** | | **Q4** | |
| --- | --- | --- | --- | --- | --- | --- | --- | --- | --- | --- |
|  | **drug** | **proportion** | **drug** | **proportion** | **drug** | **proportion** | **drug** | **proportion** | **drug** | **proportion** |
| **1** | A10BA | 42.85 (42.67-43.02) | A10BA | 45.55 (45.39-45.72) | A10BA | 46.88 (46.72-47.05) | A10BA | 47.41 (47.24-47.58) | A10BA | 45.11 (44.95-45.28) |
| **2** | A10BA A10BB | 20.74 (20.60-20.88) | A10BA A10BB | 20.82 (20.69-20.95) | A10BA A10BB | 20.27 (20.14-20.40) | A10BA A10BB | 19.87 (19.73-20.00) | A10BA A10BB | 20.31 (20.17-20.44) |
| **3** | A10A | 10.89 (10.78-11.00) | A10A | 9.07 (8.98-9.17) | A10A | 8.25 (8.16-8.34) | A10A | 7.88 (7.79-7.97) | A10A | 7.95 (7.86-8.04) |
| **4** | other | 7.72 (7.63-7.82) | other | 7.13 (7.05-7.22) | other | 7.21 (7.13-7.30) | other | 7.22 (7.13-7.31) | other | 7.37 (7.28-7.46) |
| **5** | A10BB | 6.82 (6.73-6.91) | A10BB | 6.03 (5.95-6.11) | A10BB | 5.36 (5.29-5.43) | A10BB | 4.87 (4.80-4.95) | A10A A10BA | 5.37 (5.30-5.45) |
| **6** | A10A A10BA | 3.70 (3.63-3.77) | A10A A10BA | 3.85 (3.79-3.92) | A10A A10BA | 4.12 (4.05-4.18) | A10A A10BA | 4.47 (4.40-4.54) | A10BB | 4.67 (4.60-4.74) |
| **7** | A10BA A10BB A10BG | 1.98 (1.93-2.03) | A10A A10BA A10BB | 2.20 (2.15-2.25) | A10A A10BA A10BB | 2.57 (2.51-2.62) | A10A A10BA A10BB | 2.96 (2.91-3.02) | A10A A10BA A10BB | 4.00 (3.93-4.06) |
| **8** | A10A A10BA A10BB | 1.66 (1.62-1.71) | A10BA A10BB A10BG | 1.77 (1.73-1.81) | A10BA A10BB A10BF | 1.68 (1.64-1.72) | A10BA A10BB A10BF | 1.70 (1.66-1.75) | A10BA A10BB A10BF | 1.71 (1.67-1.76) |
| **9** | A10BA A10BB A10BF | 1.55 (1.51-1.60) | A10BA A10BB A10BF | 1.61 (1.57-1.65) | A10BA A10BB A10BG | 1.68 (1.64-1.72) | A10BA A10BB A10BG | 1.58 (1.54-1.62) | A10BA A10BB A10BG | 1.37 (1.33-1.41) |
| **10** | A10BG | 1.10 (1.06-1.13) | A10BA A10BG | 0.98 (0.95-1.02) | A10BA A10BG | 1.02 (0.98-1.05) | A10BA A10BG | 1.04 (1.01-1.08) | A10BA A10BG | 1.11 (1.08-1.15) |
| **11** | A10BA A10BG | 0.98 (0.94-1.01) | A10BG | 0.98 (0.94-1.01) | A10BG | 0.97 (0.94-1.00) | A10BG | 0.99 (0.95-1.02) | A10BG | 1.02 (0.99-1.06) |
| A10A: Insulins and Analogues, A10BA: Biguanides, A10BB: Sulfonylureas, A10BD: Combinations of oral blood glucose lowering drugs, A10BF: Alpha glucosidase inhibitors, A10BG: Thiazolidinediones, A10BJ: Glucagon-like peptide-1 (GLP-1) analogues, A10BX: Other blood glucose lowering drugs, excl. insulins | | | | | | | | | | |
