# Supplementary material for: Antibiotic consumption and medication cost in diabetic patients: Insights from Iran health insurance organization (IHIO) claims data
Source: PLoS One. 2026 Feb 27;21(2):e0343090. doi: 10.1371/journal.pone.0343090 (PMC12948126; doi:10.1371/journal.pone.0343090)
Supplement: S3 Table — J01A: Tetracyclines, J01B: Amphenicols, J01C: Beta-Lactam Antibacterials, Penicillins, J01D: Other Beta-Lactam Antibacterials, J01E: Sulfonamides and Trimethoprim, J01F: Macrolides, Lincosamides and Streptogramins, J01G: Aminoglycoside Antibacterials, J01M: Quinolone Antibacterials, J01X: Other Antibacterials. (DOCX) [file pone.0343090.s003.docx]

**Supporting information**

**S3 Table. Seasonal trends in antibiotic class prescriptions (2014–2017).**

| **Antibiotic group** | **Q1** | | | | | | | | | | **Q2** | | | | | | | | | | **Q3** | | | | | | | | | | **Q4** | | | | | | | | |
| --- | --- | --- | --- | --- | --- | --- | --- | --- | --- | --- | --- | --- | --- | --- | --- | --- | --- | --- | --- | --- | --- | --- | --- | --- | --- | --- | --- | --- | --- | --- | --- | --- | --- | --- | --- | --- | --- | --- | --- |
|  | **J01A** | **J01B** | **J01C** | **J01D** | **J01E** | **J01F** | **J01G** | **J01M** | **J01X** | **J01A** | | **J01B** | **J01C** | **J01D** | **J01E** | **J01F** | **J01G** | **J01M** | **J01X** | **J01A** | | **J01B** | **J01C** | **J01D** | **J01E** | **J01F** | **J01G** | **J01M** | **J01X** | **J01A** | | **J01B** | **J01C** | **J01D** | **J01E** | **J01F** | **J01G** | **J01M** | **J01X** |
| **2014-Fall** | 803 | 4 | 22609 | 14279 | 828 | 9379 | 936 | 7223 | 201 | 2497 | | 4 | 49194 | 32567 | 1575 | 19812 | 2033 | 14369 | 523 | 5114 | | 7 | 81019 | 56583 | 2681 | 34025 | 3584 | 23925 | 1009 | 11868 | | 14 | 193011 | 147020 | 7130 | 79363 | 9988 | 53178 | 3180 |
| **2014-Spring** | 661 | 0 | 17868 | 13342 | 901 | 7809 | 979 | 7142 | 182 | 2129 | | 3 | 36792 | 28819 | 1669 | 15603 | 2038 | 13751 | 457 | 4252 | | 7 | 59168 | 47691 | 2762 | 25629 | 3343 | 22173 | 910 | 10287 | | 16 | 141087 | 123036 | 6838 | 60095 | 9172 | 49251 | 2583 |
| **2014-Summer** | 754 | 1 | 13405 | 12523 | 897 | 5335 | 979 | 7915 | 200 | 2527 | | 8 | 28582 | 27131 | 1674 | 11106 | 2124 | 15358 | 523 | 4776 | | 1 | 46105 | 45052 | 2705 | 19113 | 3778 | 24845 | 1112 | 11285 | | 6 | 116702 | 120110 | 7282 | 49418 | 9976 | 54571 | 3170 |
| **2015-Fall** | 811 | 3 | 24633 | 15734 | 844 | 11377 | 965 | 7554 | 181 | 2616 | | 7 | 53488 | 36939 | 1576 | 25377 | 2090 | 15018 | 514 | 5235 | | 11 | 86801 | 63375 | 2580 | 41873 | 3630 | 24729 | 1059 | 11640 | | 13 | 202606 | 157822 | 7078 | 93853 | 9910 | 53846 | 3337 |
| **2015-Spring** | 657 | 3 | 17741 | 13658 | 844 | 8242 | 876 | 7382 | 202 | 2546 | | 6 | 38060 | 30541 | 1505 | 17582 | 1968 | 14601 | 504 | 4916 | | 6 | 62107 | 51741 | 2498 | 29398 | 3414 | 23531 | 1000 | 12073 | | 15 | 151867 | 137454 | 7033 | 70592 | 9792 | 53496 | 3327 |
| **2015-Summer** | 893 | 9 | 13553 | 12980 | 1024 | 5614 | 946 | 8574 | 214 | 2675 | | 4 | 29449 | 29328 | 1818 | 12288 | 2223 | 16860 | 612 | 5402 | | 12 | 48812 | 47934 | 2899 | 21392 | 3622 | 27138 | 1210 | 12348 | | 16 | 127270 | 132297 | 7678 | 56941 | 10673 | 60321 | 3867 |
| **2015-Winter** | 932 | 7 | 28160 | 17334 | 815 | 13501 | 1033 | 7542 | 213 | 2839 | | 10 | 59357 | 40457 | 1473 | 28334 | 2297 | 14741 | 552 | 5723 | | 6 | 94001 | 67617 | 2562 | 46067 | 3854 | 24457 | 1073 | 13445 | | 20 | 216840 | 170876 | 7402 | 101641 | 10765 | 55411 | 3466 |
| **2016-Fall** | 733 | 0 | 22284 | 15590 | 611 | 11632 | 869 | 6862 | 205 | 2343 | | 4 | 47364 | 35388 | 1273 | 25582 | 1757 | 13322 | 537 | 4278 | | 4 | 76622 | 58774 | 2106 | 41982 | 2930 | 21136 | 1036 | 9270 | | 8 | 186960 | 147533 | 5950 | 92981 | 8336 | 47018 | 3029 |
| **2016-Spring** | 844 | 6 | 17835 | 14027 | 839 | 8808 | 911 | 7362 | 205 | 2653 | | 3 | 42134 | 34224 | 1697 | 20685 | 2118 | 15903 | 570 | 5543 | | 5 | 71594 | 60095 | 2906 | 35759 | 3677 | 26820 | 1199 | 13739 | | 5 | 183492 | 164243 | 8197 | 88136 | 10908 | 62755 | 4098 |
| **2016-Summer** | 889 | 3 | 14123 | 13559 | 887 | 6400 | 993 | 8330 | 262 | 2817 | | 1 | 32259 | 31715 | 1763 | 14913 | 2093 | 17216 | 616 | 5865 | | 1 | 55522 | 54425 | 2901 | 26099 | 3652 | 27642 | 1350 | 13841 | | 10 | 146081 | 147125 | 8084 | 68224 | 10615 | 63995 | 4313 |
| **2016-Winter** | 915 | 4 | 24905 | 16816 | 685 | 13025 | 965 | 7463 | 183 | 2897 | | 5 | 52962 | 39184 | 1315 | 27478 | 2070 | 14532 | 542 | 5491 | | 9 | 84394 | 65036 | 2285 | 45141 | 3461 | 23784 | 1211 | 12159 | | 20 | 202798 | 165841 | 6921 | 100689 | 10069 | 55017 | 3680 |
| **2017-Winter** | 805 | 0 | 19333 | 15362 | 568 | 10203 | 830 | 7052 | 220 | 2537 | | 1 | 40594 | 32929 | 1097 | 22138 | 1721 | 13175 | 559 | 4546 | | 0 | 65112 | 53339 | 1741 | 36245 | 2848 | 20796 | 1211 | 9833 | | 3 | 160828 | 134340 | 5289 | 81205 | 7742 | 45108 | 3094 |
| J01A: Tetracyclines, J01B: Amphenicols, J01C: Beta-Lactam Antibacterials, Penicillins, J01D: Other Beta-Lactam Antibacterials, J01E: Sulfonamides and Trimethoprim, J01F: Macrolides, Lincosamides and Streptogramins, J01G: Aminoglycoside Antibacterials, J01M: Quinolone Antibacterials, J01X: Other Antibacterials | | | | | | | | | | | | | | | | | | | | | | | | | | | | | | | | | | | | | | | |
