# Supplementary material for: Antibiotic consumption and medication cost in diabetic patients: Insights from Iran health insurance organization (IHIO) claims data
Source: PLoS One. 2026 Feb 27;21(2):e0343090. doi: 10.1371/journal.pone.0343090 (PMC12948126; doi:10.1371/journal.pone.0343090)
Supplement: S4 Table — (DOCX) [file pone.0343090.s004.docx]

**Supporting information**

**S4 Table. Median annual costs by province.**

|  | **Antibiotics** | | **Glucose lowering drugs** | | **Others** | | **Total** |
| --- | --- | --- | --- | --- | --- | --- | --- |
| **Province** | **Out of pocket** | **Total** | **Out of pocket** | **Total** | **Out of pocket** | **Total** |  |
| **Markazi** | 0.64 (1.36) | 2.15 (4.53) | 0.93 (2.79) | 3.62 (12.84) | 4.80 (10.17) | 16.56 (35.77) | 29.62 (59.89) |
| **Gilan** | 0.67 (1.47) | 2.23 (4.90) | 1.33 (4.87) | 5.31 (21.24) | 5.46 (11.47) | 18.83 (40.51) | 35.79 (78.06) |
| **Mazandaran** | 0.80 (2.15) | 2.01 (4.53) | 1.78 (7.12) | 5.22 (20.56) | 7.05 (15.21) | 16.20 (34.12) | 32.67 (66.89) |
| **Kermanshah** | 0.93 (1.81) | 3.09 (6.04) | 0.76 (2.13) | 2.65 (9.23) | 4.61 (8.68) | 16.02 (30.79) | 28.99 (51.83) |
| **Fars** | 0.33 (0.83) | 2.31 (4.30) | 0.45 (1.63) | 4.32 (16.05) | 2.60 (6.08) | 16.46 (33.72) | 31.39 (60.47) |
| **Kerman** | 0.53 (1.15) | 1.65 (3.47) | 1.12 (5.12) | 3.93 (17.65) | 4.48 (9.64) | 14.07 (29.45) | 27.72 (59.68) |
| **Khorasan, Razavi** | 0.58 (1.23) | 1.94 (4.11) | 0.96 (3.04) | 3.89 (13.29) | 4.52 (9.34) | 15.68 (33.24) | 28.76 (58.33) |
| **Isfahan** | 0.69 (1.29) | 2.29 (4.31) | 0.97 (3.90) | 3.75 (16.98) | 5.35 (10.59) | 18.46 (37.64) | 33.15 (69.29) |
| **Sistan and Baluchestan** | 0.86 (1.54) | 2.84 (5.12) | 0.66 (1.93) | 2.58 (9.33) | 3.73 (6.89) | 12.60 (23.87) | 24.09 (42.54) |
| **Kurdistan** | 0.67 (1.22) | 2.24 (4.06) | 0.80 (2.09) | 2.87 (10.17) | 3.48 (7.09) | 12.12 (25.21) | 23.41 (45.28) |
| **Hamadan** | 0.64 (1.44) | 2.13 (4.80) | 0.80 (2.54) | 3.01 (11.20) | 3.89 (8.39) | 13.36 (29.52) | 26.18 (54.47) |
| **Chaharmahal and Bakhtiari** | 0.72 (1.55) | 2.40 (5.15) | 0.72 (2.28) | 2.65 (9.91) | 5.32 (10.91) | 18.18 (38.10) | 30.67 (60.68) |
| **Lorestan** | 0.78 (1.68) | 2.60 (5.57) | 0.66 (1.90) | 2.52 (8.80) | 4.30 (8.46) | 14.88 (29.99) | 26.67 (50.76) |
| **Ilam** | 1.80 (3.04) | 5.92 (9.96) | 0.68 (1.99) | 2.61 (7.70) | 7.70 (12.74) | 24.53 (41.59) | 40.89 (63.89) |
| **Kohgiluyeh and Boyer-Ahmad** | 1.28 (2.24) | 4.25 (7.46) | 0.76 (1.78) | 2.65 (8.46) | 5.89 (10.01) | 20.07 (34.74) | 34.95 (55.66) |
| **Bushehr** | 0.65 (1.35) | 2.18 (4.49) | 0.86 (2.93) | 3.75 (14.95) | 4.03 (8.23) | 13.92 (29.24) | 27.37 (55.95) |
| **Zanjan** | 0.52 (1.13) | 1.73 (3.75) | 1.13 (4.50) | 4.69 (21.21) | 4.21 (8.95) | 14.52 (31.79) | 30.75 (65.41) |
| **Yazd** | 0.57 (1.13) | 1.91 (3.76) | 1.31 (5.32) | 5.16 (24.71) | 4.63 (9.56) | 15.99 (33.74) | 32.50 (74.10) |
| **Hormozgan** | 0.41 (1.11) | 1.37 (3.68) | 0.80 (2.33) | 3.06 (11.12) | 2.42 (5.45) | 8.27 (19.06) | 18.71 (39.67) |
| **Tehran** | 0.54 (1.10) | 1.80 (3.65) | 1.15 (3.56) | 4.63 (16.48) | 4.30 (9.06) | 14.80 (32.23) | 29.04 (61.09) |
| **Qazvin** | 0.57 (1.15) | 1.90 (3.82) | 1.05 (3.33) | 4.25 (15.46) | 3.81 (8.07) | 13.27 (28.34) | 27.01 (54.53) |
| **Golestan** | 0.95 (2.21) | 3.16 (7.38) | 0.81 (2.57) | 3.30 (12.24) | 5.01 (10.44) | 17.27 (36.80) | 32.79 (67.86) |
| **Khorasan, North** | 0.81 (1.67) | 2.69 (5.58) | 0.67 (1.98) | 2.56 (8.55) | 4.19 (8.71) | 14.32 (30.43) | 25.93 (52.03) |
| **Khorasan, South** | 0.67 (1.36) | 2.23 (4.52) | 0.90 (2.86) | 3.55 (12.72) | 4.40 (8.81) | 15.15 (31.23) | 28.05 (54.11) |
