# Supplementary material for: Antibiotic consumption and medication cost in diabetic patients: Insights from Iran health insurance organization (IHIO) claims data
Source: PLoS One. 2026 Feb 27;21(2):e0343090. doi: 10.1371/journal.pone.0343090 (PMC12948126; doi:10.1371/journal.pone.0343090)
Supplement: S5 Table — (DOCX) [file pone.0343090.s005.docx]

**Supporting information**

**S5 Table. Mean annual costs by province.**

| **Province** | **Antibiotics** | | **Glucose lowering drugs** | | **Others** | | **Total** |
| --- | --- | --- | --- | --- | --- | --- | --- |
|  | **Percentage paid out of pocket** | **Percentage of total cost** | **Percentage paid out of pocket** | **Percentage of total cost** | **Percentage paid out of pocket** | **Percentage of total cost** |  |
| **Markazi** | 30.05 (29.57-30.53) | 4.46 (4.39-4.53) | 14.57 (14.17-14.97) | 33.28 (32.12-34.45) | 21.45 (20.34-22.57) | 62.26 (58.25-66.27) | 88 (84-92) |
| **Gilan** | 30.03 (29.69-30.38) | 3.83 (3.79-3.88) | 14.66 (14.38-14.95) | 44.95 (43.82-46.07) | 22.54 (21.81-23.26) | 51.22 (49.41-53.03) | 120 (117-122) |
| **Mazandaran** | 46.47 (45.96-46.99) | 4.06 (4.02-4.10) | 19.74 (19.46-20.02) | 46.79 (45.78-47.80) | 32.02 (31.35-32.69) | 49.15 (47.46-50.83) | 99 (97-100) |
| **Kermanshah** | 30.00 (29.65-30.34) | 7.38 (7.29-7.46) | 15.80 (15.38-16.22) | 32.61 (31.52-33.70) | 23.05 (22.24-23.86) | 60.01 (57.54-62.49) | 76 (73-78) |
| **Fars** | 16.66 (16.51-16.81) | 4.19 (4.16-4.22) | 8.63 (8.48-8.77) | 38.49 (37.84-39.13) | 15.83 (15.41-16.25) | 57.32 (55.84-58.79) | 93 (92-95) |
| **Kerman** | 33.35 (32.93-33.78) | 2.61 (2.58-2.65) | 15.47 (15.13-15.81) | 51.96 (50.53-53.38) | 24.62 (23.42-25.82) | 45.43 (42.96-47.89) | 114 (110-117) |
| **Khorasan, Razavi** | 29.93 (29.62-30.24) | 4.09 (4.04-4.13) | 13.96 (13.77-14.15) | 38.74 (38.09-39.39) | 21.75 (21.25-22.25) | 57.17 (55.57-58.77) | 93 (91-94) |
| **Isfahan** | 29.99 (29.75-30.24) | 3.50 (3.48-3.53) | 13.73 (13.54-13.92) | 42.80 (42.06-43.53) | 22.17 (21.58-22.77) | 53.70 (52.19-55.21) | 112 (110-114) |
| **Sistan and Baluchestan** | 30.17 (29.87-30.46) | 6.24 (6.18-6.30) | 14.68 (14.23-15.13) | 45.60 (44.07-47.14) | 23.17 (22.16-24.17) | 48.15 (45.92-50.39) | 72 (70-74) |
| **Kurdistan** | 29.98 (29.63-30.33) | 5.00 (4.94-5.06) | 14.32 (13.90-14.74) | 39.49 (38.02-40.97) | 21.58 (20.84-22.31) | 55.51 (52.84-58.17) | 71 (69-74) |
| **Hamadan** | 30.07 (29.54-30.60) | 4.78 (4.70-4.87) | 14.07 (13.67-14.48) | 40.50 (39.09-41.91) | 21.93 (20.86-23.00) | 54.71 (51.63-57.80) | 90 (86-93) |
| **Chaharmahal and Bakhtiari** | 30.05 (29.51-30.59) | 4.72 (4.63-4.80) | 14.64 (14.01-15.28) | 33.81 (31.94-35.69) | 22.83 (21.45-24.21) | 61.47 (56.91-66.02) | 92 (88-97) |
| **Lorestan** | 30.13 (29.72-30.55) | 7.05 (6.95-7.14) | 14.65 (14.23-15.07) | 34.82 (33.55-36.08) | 24.01 (23.18-24.83) | 58.14 (55.43-60.84) | 69 (67-71) |
| **Ilam** | 30.53 (30.10-30.95) | 10.84 (10.69-10.99) | 17.16 (16.50-17.83) | 25.16 (23.79-26.53) | 25.81 (24.81-26.81) | 64.00 (60.70-67.30) | 83 (80-86) |
| **Kohgiluyeh and Boyer-Ahmad** | 30.01 (29.56-30.45) | 8.16 (8.04-8.28) | 14.73 (13.93-15.52) | 36.93 (34.59-39.26) | 25.47 (24.07-26.87) | 54.91 (51.34-58.49) | 79 (75-82) |
| **Bushehr** | 30.12 (29.43-30.80) | 4.17 (4.09-4.25) | 13.67 (13.10-14.25) | 45.94 (43.60-48.27) | 20.67 (19.71-21.62) | 49.89 (46.35-53.43) | 91 (87-95) |
| **Zanjan** | 30.03 (29.12-30.93) | 2.81 (2.72-2.89) | 14.04 (13.45-14.63) | 47.00 (44.58-49.42) | 20.31 (18.55-22.07) | 50.20 (44.49-55.90) | 117 (110-125) |
| **Yazd** | 30.20 (29.75-30.65) | 2.48 (2.45-2.52) | 12.85 (12.48-13.22) | 57.77 (55.87-59.68) | 21.77 (20.66-22.88) | 39.74 (37.56-41.93) | 131 (127-134) |
| **Hormozgan** | 30.09 (29.53-30.65) | 3.35 (3.29-3.41) | 14.06 (13.41-14.70) | 50.27 (47.77-52.77) | 20.57 (18.73-22.40) | 46.38 (41.82-50.95) | 81 (77-85) |
| **Tehran** | 30.10 (29.87-30.33) | 2.95 (2.93-2.98) | 14.02 (13.88-14.16) | 35.09 (34.66-35.53) | 20.36 (19.87-20.86) | 61.95 (60.37-63.53) | 112 (110-114) |
| **Qazvin** | 30.12 (29.42-30.82) | 3.59 (3.51-3.67) | 14.07 (13.51-14.63) | 39.99 (37.97-42.00) | 20.60 (18.65-22.56) | 56.43 (50.54-62.31) | 95 (89-101) |
| **Golestan** | 29.96 (29.51-30.41) | 6.87 (6.76-6.97) | 13.10 (12.77-13.42) | 37.61 (36.51-38.71) | 21.93 (21.08-22.78) | 55.53 (53.14-57.91) | 101 (99-104) |
| **Khorasan, North** | 29.98 (29.36-30.59) | 6.96 (6.82-7.11) | 14.25 (13.58-14.91) | 39.25 (36.91-41.58) | 24.69 (22.95-26.43) | 53.79 (49.25-58.33) | 74 (70-78) |
| **Khorasan, South** | 30.00 (29.37-30.62) | 4.71 (4.61-4.81) | 14.19 (13.54-14.83) | 43.29 (40.81-45.78) | 22.64 (21.17-24.11) | 51.99 (47.70-56.29) | 84 (79-88) |
