# Supplementary material for: Antibiotic consumption and medication cost in diabetic patients: Insights from Iran health insurance organization (IHIO) claims data
Source: PLoS One. 2026 Feb 27;21(2):e0343090. doi: 10.1371/journal.pone.0343090 (PMC12948126; doi:10.1371/journal.pone.0343090)
Supplement: S6 Table — Reference values of variables are: 18−39 for age group, No antibiotic for antibiotic group, A10BA for dominant diabetes treatment regimen, rural for fund, female for sex, and Tehran for province. A10A: Insulins and Analogues, A10BA: Biguanides, A10BB: Sulfonylureas, A10BD: Combinations of oral blood glucose lowering drugs, A10BF: Alpha glucosidase inhibitors, A10BG: Thiazolidinediones, A10BJ: Glucagon-like peptide-1 (GLP-1) analogues, A10BX: Other blood glucose lowering drugs, excl. insulins. (DOCX) [file pone.0343090.s006.docx]

**Supporting information**

**S6 Table. Gamma Generalized linear model (GLM) adjusted mean ratios for total medication costs.**

| **Variable** | **Value** | **Mean ratio** | **P value** |
| --- | --- | --- | --- |
| **Sex** | **Male** | 0.98 (0.96-0.99) | 0.003 |
| **Fund** | **Civil servants** | 1.51 (1.48-1.55) | <0.001 |
|  | **Iranian** | 1.51 (1.47-1.55) | <0.001 |
|  | **Universal** | 0.72 (0.70-0.74) | <0.001 |
|  | **Foreign** | 0.63 (0.51-0.77) | <0.001 |
|  | **Others** | 1.41 (1.37-1.46) | <0.001 |
| **Age group** | **40-65** | 1.33 (1.31-1.36) | <0.001 |
|  | **65-95** | 1.50 (1.47-1.54) | <0.001 |
| **Antibiotic group** | **Q1** | 1.39 (1.36-1.42) | <0.001 |
|  | **Q2** | 1.71 (1.67-1.75) | <0.001 |
|  | **Q3** | 2.12 (2.07-2.17) | <0.001 |
|  | **Q4** | 3.17 (3.09-3.25) | <0.001 |
| **Province** | **Bushehr** | 0.77 (0.72-0.82) | <0.001 |
|  | **Chaharmahal and Bakhtiari** | 0.83 (0.78-0.89) | <0.001 |
|  | **Fars** | 0.92 (0.90-0.95) | <0.001 |
|  | **Gilan** | 0.99 (0.96-1.03) | 0.676 |
|  | **Golestan** | 0.75 (0.72-0.78) | <0.001 |
|  | **Hamadan** | 0.82 (0.78-0.86) | <0.001 |
|  | **Hormozgan** | 0.68 (0.64-0.72) | <0.001 |
|  | **Ilam** | 0.63 (0.59-0.67) | <0.001 |
|  | **Isfahan** | 0.89 (0.87-0.92) | <0.001 |
|  | **Kerman** | 0.89 (0.86-0.93) | <0.001 |
|  | **Kermanshah** | 0.75 (0.72-0.78) | <0.001 |
|  | **Khorasan, North** | 0.67 (0.63-0.72) | <0.001 |
|  | **Khorasan, Razavi** | 0.83 (0.81-0.85) | <0.001 |
|  | **Khorasan, South** | 0.70 (0.65-0.75) | <0.001 |
|  | **Kohgiluyeh and Boyer-Ahmad** | 0.63 (0.59-0.67) | <0.001 |
|  | **Kurdistan** | 0.71 (0.68-0.74) | <0.001 |
|  | **Lorestan** | 0.66 (0.63-0.69) | <0.001 |
|  | **Markazi** | 0.81 (0.76-0.85) | <0.001 |
|  | **Mazandaran** | 0.78 (0.75-0.81) | <0.001 |
|  | **Qazvin** | 0.85 (0.79-0.91) | <0.001 |
|  | **Sistan and Baluchestan** | 0.62 (0.60-0.65) | <0.001 |
|  | **Yazd** | 0.90 (0.85-0.95) | <0.001 |
|  | **Zanjan** | 1.08 (1.00-1.16) | 0.044 |
| **Dominant diabetes treatment regimen** | **A10A** | 5.53 (5.38-5.68) | <0.001 |
|  | **A10A A10BA** | 5.75 (5.54-5.97) | <0.001 |
|  | **A10A A10BA A10BB** | 3.78 (3.61-3.96) | <0.001 |
|  | **A10BA A10BB** | 1.08 (1.06-1.11) | <0.001 |
|  | **A10BA A10BB A10BF** | 1.81 (1.71-1.92) | <0.001 |
|  | **A10BA A10BB A10BG** | 1.65 (1.55-1.74) | <0.001 |
|  | **A10BA A10BG** | 1.43 (1.33-1.54) | <0.001 |
|  | **A10BB** | 0.92 (0.89-0.95) | <0.001 |
|  | **A10BG** | 1.26 (1.17-1.36) | <0.001 |
|  | **other** | 2.32 (2.25-2.39) | <0.001 |
| Reference values of variables are: 18-39 for age group, No antibiotic for antibiotic group, A10BA for dominant diabetes treatment regimen, rural for fund, female for sex, and Tehran for province.  A10A: Insulins and Analogues, A10BA: Biguanides, A10BB: Sulfonylureas, A10BD: Combinations of oral blood glucose lowering drugs, A10BF: Alpha glucosidase inhibitors, A10BG: Thiazolidinediones, A10BJ: Glucagon-like peptide-1 (GLP-1) analogues, A10BX: Other blood glucose lowering drugs, excl. insulins | | | |
