# Supplementary material for: Antibiotic consumption and medication cost in diabetic patients: Insights from Iran health insurance organization (IHIO) claims data
Source: PLoS One. 2026 Feb 27;21(2):e0343090. doi: 10.1371/journal.pone.0343090 (PMC12948126; doi:10.1371/journal.pone.0343090)
Supplement: S7 Table — Reference values of variables are: 18−39 for age group, No antibiotic for antibiotic group, A10BA for dominant diabetes treatment regimen, rural for fund, female for sex, and Tehran for province. A10A: Insulins and Analogues, A10BA: Biguanides, A10BB: Sulfonylureas, A10BD: Combinations of oral blood glucose lowering drugs, A10BF: Alpha glucosidase inhibitors, A10BG: Thiazolidinediones, A10BJ: Glucagon-like peptide-1 (GLP-1) analogues, A10BX: Other blood glucose lowering drugs, excl. insulins. (DOCX) [file pone.0343090.s007.docx]

**Supporting information**

**S7 Table.** **Gamma Generalized linear model (GLM) crude mean ratios for total medication costs.**

| **Variable** | **Value** | **Mean ratio** | **P value** |
| --- | --- | --- | --- |
| **Sex** | **Male** | 1.16 (1.14-1.17) | <0.001 |
| **Fund** | **Civil servants** | 1.93 (1.90-1.96) | <0.001 |
|  | **Iranian** | 1.84 (1.80-1.87) | <0.001 |
|  | **Universal** | 0.68 (0.67-0.69) | <0.001 |
|  | **Foreign** | 0.60 (0.51-0.70) | <0.001 |
|  | **Others** | 1.90 (1.86-1.94) | <0.001 |
| **Age group** | **40-65** | 1.77 (1.75-1.80) | <0.001 |
|  | **65-95** | 2.09 (2.05-2.13) | <0.001 |
| **Antibiotic group** | **Q1** | 1.36 (1.33-1.38) | <0.001 |
|  | **Q2** | 1.68 (1.65-1.71) | <0.001 |
|  | **Q3** | 2.07 (2.03-2.11) | <0.001 |
|  | **Q4** | 3.08 (3.02-3.14) | <0.001 |
| **Province** | **Bushehr** | 0.82 (0.78-0.86) | <0.001 |
|  | **Chaharmahal and Bakhtiari** | 0.83 (0.79-0.87) | <0.001 |
|  | **Fars** | 0.83 (0.82-0.85) | <0.001 |
|  | **Gilan** | 1.07 (1.04-1.10) | <0.001 |
|  | **Golestan** | 0.91 (0.88-0.94) | <0.001 |
|  | **Hamadan** | 0.80 (0.77-0.83) | <0.001 |
|  | **Hormozgan** | 0.72 (0.69-0.76) | <0.001 |
|  | **Ilam** | 0.74 (0.71-0.78) | <0.001 |
|  | **Isfahan** | 1.00 (0.98-1.03) | 0.681 |
|  | **Kerman** | 1.02 (0.98-1.05) | 0.344 |
|  | **Kermanshah** | 0.68 (0.65-0.70) | <0.001 |
|  | **Khorasan, North** | 0.67 (0.63-0.70) | <0.001 |
|  | **Khorasan, Razavi** | 0.83 (0.81-0.85) | <0.001 |
|  | **Khorasan, South** | 0.75 (0.71-0.79) | <0.001 |
|  | **Kohgiluyeh and Boyer-Ahmad** | 0.70 (0.67-0.74) | <0.001 |
|  | **Kurdistan** | 0.64 (0.62-0.66) | <0.001 |
|  | **Lorestan** | 0.61 (0.59-0.64) | <0.001 |
|  | **Markazi** | 0.79 (0.75-0.82) | <0.001 |
|  | **Mazandaran** | 0.88 (0.86-0.91) | <0.001 |
|  | **Qazvin** | 0.85 (0.80-0.90) | <0.001 |
|  | **Sistan and Baluchestan** | 0.64 (0.62-0.67) | <0.001 |
|  | **Yazd** | 1.17 (1.12-1.22) | <0.001 |
|  | **Zanjan** | 1.05 (0.99-1.11) | 0.088 |
| **Dominant diabetes treatment regimen** | **A10A** | 5.05 (4.91-5.19) | <0.001 |
|  | **A10A A10BA** | 6.24 (6.01-6.48) | <0.001 |
|  | **A10A A10BA A10BB** | 4.39 (4.19-4.60) | <0.001 |
|  | **A10BA A10BB** | 1.21 (1.19-1.24) | <0.001 |
|  | **A10BA A10BB A10BF** | 1.98 (1.86-2.10) | <0.001 |
|  | **A10BA A10BB A10BG** | 1.72 (1.62-1.83) | <0.001 |
|  | **A10BA A10BG** | 1.54 (1.43-1.66) | <0.001 |
|  | **A10BB** | 0.98 (0.95-1.02) | 0.369 |
|  | **A10BG** | 1.30 (1.20-1.40) | <0.001 |
|  | **other** | 2.45 (2.38-2.53) | <0.001 |
| Reference values of variables are: 18-39 for age group, No antibiotic for antibiotic group, A10BA for dominant diabetes treatment regimen, rural for fund, female for sex, and Tehran for province.  A10A: Insulins and Analogues, A10BA: Biguanides, A10BB: Sulfonylureas, A10BD: Combinations of oral blood glucose lowering drugs, A10BF: Alpha glucosidase inhibitors, A10BG: Thiazolidinediones, A10BJ: Glucagon-like peptide-1 (GLP-1) analogues, A10BX: Other blood glucose lowering drugs, excl. insulins | | | |
