# Supplementary material for: Antibiotic consumption and medication cost in diabetic patients: Insights from Iran health insurance organization (IHIO) claims data
Source: PLoS One. 2026 Feb 27;21(2):e0343090. doi: 10.1371/journal.pone.0343090 (PMC12948126; doi:10.1371/journal.pone.0343090)
Supplement: Attachment — Submitted filename: Response_to_Reviewers_auresp_2.docx [file pone.0343090.s009.docx]

**Response to Reviewers**

**Authors:** We thank the Academic Editor and Reviewers for their careful evaluation of our manuscript and for the constructive comments provided. We have revised the manuscript thoroughly to address each point raised.

Editorial Requirements

References should be typed according to the PLoS ONE guidelines.

**Response:** Thank you for your feedback. Your comment is considered.

**Sincerely,**

**Authors**
